# Dehydrin expression in seeds: an issue of maturation drying

**DOI:** 10.3389/fpls.2014.00402

**Published:** 2014-08-27

**Authors:** Maik Kleinwächter, Alzahraa Radwan, Masakazu Hara, Dirk Selmar

**Affiliations:** ^1^Institute for Plant Biology, Department of Life SciencesTechnische Universität Braunschweig, Germany; ^2^Laboratory of Functional Plant Physiology, Faculty of Agriculture, Shizuoka UniversityShizuoka, Japan

**Keywords:** dehydrin, maturation drying, orthodox seeds, recalcitrant seeds, drought stress

## The ecological relevance of maturation drying determines dehydrin expression in seeds

In areas exhibiting typical seasonal changes, the seeds of most plants are getting mature in summer and fall, respectively. In order to prevent the destruction of the young and sensitive seedlings by the frosty winter conditions, any germination in fall has to be hindered. Accordingly, efficient outlasting mechanisms have been evolved to avoid or at least to minimize such wastages by suppressing the germination of mature seeds in the fall and allow the seeds to overwinter without any loss of their viability (Bewley, 1997). Yet, such persistence requires strongly reduced metabolic activity, i.e., by the reduction of the water content. Accordingly, apart from the suppression of germination, desiccation tolerance is a precondition for the overwintering of the seeds (for review see Finch-Savage and Leubner-Metzger, 2006). In this manner, during the last phase of embryogenesis, the water content of the seeds is strongly reduced in a process denoted as maturation drying. As result the seeds become dormant. In the succeeding spring, i.e., after a prolonged cold period, dormancy is broken (vernalization) and seeds are considered as quiescent (Baskin and Baskin, 2004). As soon as sufficient water is available, the quiescent seeds imbibe and germinate. In contrast to such orthodox seeds, seeds of most tropical plants are able to germinate as soon as the fruits are mature (for review see Farnsworth, 2000). In tropical rainforests there are no unfavorable climatic conditions that have to be overcome, since there is no necessity for putative outlasting mechanisms. In contrast, any extension of the exposition of ungerminated seeds would enhance the risk of being eaten by herbivores or being infected by pathogens. Accordingly, the seeds of most tropical plants have not evolved outlasting mechanisms, and, most tropical seeds are recalcitrant: Dormancy is lacking (e.g., Jurado and Flores, 2005) and they do not reveal a maturation drying (Berjak et al., 1989). As a result, recalcitrant seeds cannot be stored for a long period of time without losing their viability. The most popular plants that exhibit recalcitrant seeds are: avocado, cocoa, mango, lychee, and the rubber tree.

Indeed, these ecological cognitions have been well known for several decades. Yet, due to the fact that recalcitrant seeds are not storable, in many scientific articles—especially those focusing on breeding, seed production and seed storage—recalcitrance *per se* is considered a negative property (e.g., Farnsworth, 2000). In consequence, many efforts are made to overcome the apparent drawbacks of recalcitrance by improving the seed storability of plants used in agronomy.

## Maturation drying corresponds to drought stress of seeds

Just as the breeders, who—due to their particular perspectives—generally focus on only one aspect of recalcitrance, also molecular biologists frequently exhibit a restricted cognition of recalcitrance and maturation drying. This, in particular, applies for the assessment of dehydrins and their relevance with respect to the storage of recalcitrant seeds. Accordingly, frequently it is stated that “*the lack of desiccation tolerance might be related to the absence of dehydrins*” (e.g., Farrant et al., 1996; Hundertmark et al., 2011). There is no doubt that dehydrins, jointly with other protective mechanisms, such as other LEA proteins are relevant for the desiccation tolerance and storability of vital seeds (Delahaie et al., 2013). However, we have to omit confounding cause and effect. Commonly in scientific articles it is outlined that “*LEA proteins (and thus dehydrins) are intensively synthesized during seed development as a part of the embryogenesis program*” (e.g., Kalemba and Pukacka, 2007). But, it is the occurrence of the maturation drying, which in particular induces the various drought stress related reactions, such as the expression of dehydrins. When maturation drying is lacking, the corresponding stress responses are not induced and the protective mechanisms are missing. In consequence, the seeds are desiccation sensitive and loose their viability when stored for a longer time. In contrast, seeds which have undergone a maturation drying are desiccation tolerant and are enabled to remain vital whilst storage.

**Figure 1:**
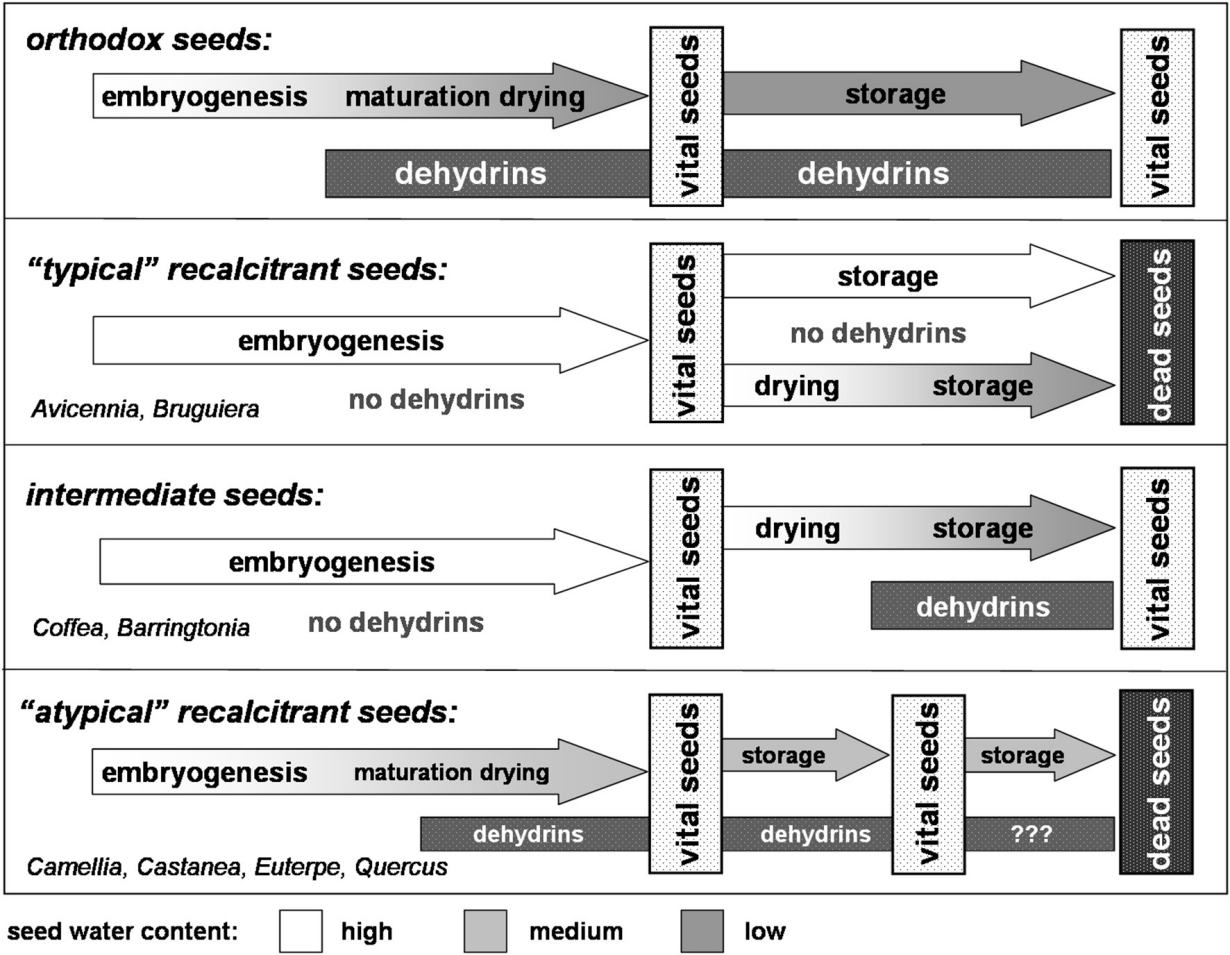
**Dehydrin expression and maturation drying–An adjustment to the chain of events (according to Radwan et al., 2014)**.

The basic mechanisms occurring in seeds undergoing a maturation drying, in principle, are the same as in leaves exposed to drought stress. In leaves subjected to various stress situations, particularly to drought stress, dehydrins are synthesized as part of the protective response (for review see Close, 1997; Hara, 2010). Consequently, the occurrence of dehydrins in seeds undergoing a maturation drying has to be considered as a direct response to drought stress. Although the direct mode of action of dehydrins with respect to drought resistance has not yet been unequivocally proved, their fundamental relevance is out of question (for review see Hara, 2010). As outlined above, alike to the well-known stress responses in leaves, also the corresponding water shortage during maturation drying induces the various protective mechanisms, such as the production of dehydrins. These coherences become particularly obvious when comparing the dehydrin expression in orthodox and recalcitrant seeds (Figure 1). Whereas orthodox seeds—as response to the maturation drying—synthesize dehydrins, in recalcitrant seeds these small protective proteins are lacking, putatively due to the absence of any maturation drying (e.g., *Avicennia marina, Bruguiera cylindrical;* Farrant et al., 1992, 1996). In consequence orthodox seeds retain their viability during storage, and recalcitrant ones die.

These insights become even more apparent, when focusing on so-called intermediate seeds, e.g., those of the coffee tree (*Coffea arabica*, Figure 1). Like recalcitrant seeds, coffee seeds do not undergo a maturation drying. In consequence, no dehydrins are expressed. However, like orthodox seeds, coffee seeds could be dried without losing their viability, e.g., during the course of green coffee processing. It turned out that directly after the onset of drying, dehydrins are expressed and synthesized (Kramer et al., 2010). As consequence of the dehydrin accumulation—in concert with various other protective mechanisms—seeds acquire desiccation tolerance. Accordingly, dried coffee seeds can be stored without losing their viability (Selmar et al., 2008). A corresponding drying related induction of dehydrin expression was reported for the seeds of *Barringtonia racemosa* (Farrant et al., 1996). In this context we have to consider that—in contrast to many citations classifying *Barringtonia* seeds as recalcitrant—these seeds have to be denoted as intermediate: they are able to withstand artificial drying and remain viable for many months (Baskin and Baskin, 2001).

On a first glance, the situation becomes much more confusing and seems to be contradictory, when considering various recalcitrant plants, whose seeds are reported to express dehydrins, e.g., *Castanea sativa* (Finch-Savage et al., 1994), *Aesculus hippocastaneum, Camellia sinensis* (Farrant et al., 1996), *Castanospermum australe* (Han et al., 1997), *Euterpe edulis* (Panza et al., 2007), and *Quercus robur* (Šunderlíková et al., 2009). However, a detailed analysis of the particular situation reveals that also in these cases the order of events also matches the scheme shown in Figure 1: the seeds of theses atypical recalcitrant plants, e.g., those from *Quercus robur; Euterpe edulis*, indeed undergo a faint maturation drying (e.g., Finch-Savage et al., 1992). The corresponding water loss obviously is sufficient to induce dehydrin expression and other stress responses. In consequence, the partially dried seeds can be stored for a limited time, e.g., to outlast the forthcoming winter season. However, this storability is limited and any longer storage of these recalcitrant seeds results in the loss of their viability.

Apart from stress related dehydrin expression, certain types of dehydrins might also be expressed constitutively, acting as some kind of house-keeping genes, too (Hara et al., 2011). In this manner, Šunderlíková et al. (2009) reported that apart from the YnSKn type, which is expressed in response to osmotic or desiccation stress in oak embryos, two Kn type dehydrins are expressed constitutively. Moreover, dehydrins are thought to be involved in developmental processes, such as germination (Gumilevskaya and Azarkovich, 2010), and their expression is related to organ type and developmental stage (e.g., Vaseva et al., 2014).

Based on the insights expounded in this article, many putative discrepancies in the perceptions of maturation drying, storability, and recalcitrance should be eliminated. Nonetheless, there are still many questions to be answered and comprehensive and comparative studies are required. Special emphasis should be put on dehydrin expression, its accumulation and the corresponding regulation in recalcitrant, intermediate, and orthodox seeds whilst maturation drying, and in leaves facing drought stress.

### Conflict of interest statement

The authors declare that the research was conducted in the absence of any commercial or financial relationships that could be construed as a potential conflict of interest.
